# Exome sequencing reveals a novel *PLP1* mutation in a Moroccan family with connatal Pelizaeus-Merzbacher disease: a case report

**DOI:** 10.1186/s12887-018-1063-5

**Published:** 2018-02-27

**Authors:** Jaber Lyahyai, Bouchra Oulad Amar Bencheikh, Siham C. Elalaoui, Maria Mansouri, Lamia Boualla, Alexandre DIonne-Laporte, Dan Spiegelman, Patrick A. Dion, Patrick Cossette, Guy A. Rouleau, Abdelaziz Sefiani

**Affiliations:** 1 Centre de Génomique Humaine, Faculté de Médecine et Pharmacie, Mohammed V University in Rabat, Rabat, Morocco; 2 Montreal Neurological Institute and Hospital, Montreal, Quebec, Canada; 3 Department of Neurology and Neurosurgery, McGill University, Montreal, Quebec Canada; 4 Département de Génétique Médicale, Institut National d’Hygiène, Rabat, Morocco; 5 Molecular Diagnostic Laboratory and Division of Medical Genetics, CHU Sainte-Justine, Montreal, Quebec, Canada

**Keywords:** Missense mutation, *PLP1* gene, Connatal Pelizaeus-Merzbacher disease

## Abstract

**Background:**

Epilepsy regroups a common and diverse set of chronic neurological disorders that are characterized by spontaneous, unprovoked, and recurrent epileptic seizures. Epilepsies have a highly heterogeneous background with a strong genetic contribution and various mode of inheritance. X-linked epilepsy usually manifests as part of a syndrome or epileptic encephalopathy. The variability of clinical manifestations of X-linked epilepsy may be attributed to several factors including the causal genetic mutation, making diagnosis, genetic counseling and treatment decisions difficult. We report the description of a Moroccan family referred to our genetic department with X-linked epileptic seizures as the only initial diagnosis.

**Case presentation:**

Knowing the new contribution of Next-Generation Sequencing (NGS) for clinical investigation, and given the heterogeneity of this group of disorders we performed a Whole-Exome Sequencing (WES) analysis and co-segregation study in several members of this large family. We detected a novel pathogenic *PLP1* missense mutation c.251C > A (p.Ala84Asp) allowing us to make a diagnosis of Pelizaeus-Merzbacher Disease for this family.

**Conclusion:**

This report extends the spectrum of *PLP1* mutations and highlights the diagnostic utility of NGS to investigate this group of heterogeneous disorders.

## Background

Epilepsy regroups a large group of highly heterogeneous disorders that have in common an abnormally increased predisposition to seizures. Epilepsy is defined as a common and diverse set of neurologic disorders characterized by spontaneous, unprovoked and recurrent epileptic seizures [1].

Multiple genetic factors were identified to be involved in primary epilepsy syndromes for which epileptic seizures are the predominant clinical feature; the same hold true in brain developmental disorders for which epileptic seizures are secondary clinical features. Recent whole-exome sequencing (WES) data suggest that mutations causing epileptic encephalopathies are often sporadic. These mutations are typically de novo dominant variations that affect a single autosomal gene but autosomal recessive and X-linked inheritance were also reported [2].

Generally the X-linked epileptic forms orient to a group of disorders which, among others, consist of: Epilepsy, X-linked, with Variable Learning Disabilities and Behavior Disorders (OMIM #300491), Epileptic Encephalopathy, Early Infantile, type 1 (EIEE1; OMIM #308350) and type 2 (EIEE2; OMIM #300672), and rarely Pelizaeus-Merzbacher Disease (PMD; OMIM #312080) [3–5].

We report here a family addressed to our department with an initial diagnosis of X-linked epileptic seizures. Given the heterogeneity of this group of diseases we used a whole-exome sequencing approach that allowed us to detect a novel *PLP1* missense mutation p.Ala84Asp related to PMD. This diagnosis was consistent with the phenotype of the affected patients, and confirmed by segregation analysis of the mutation in the family.

## Case presentation

**Fig. 1:**
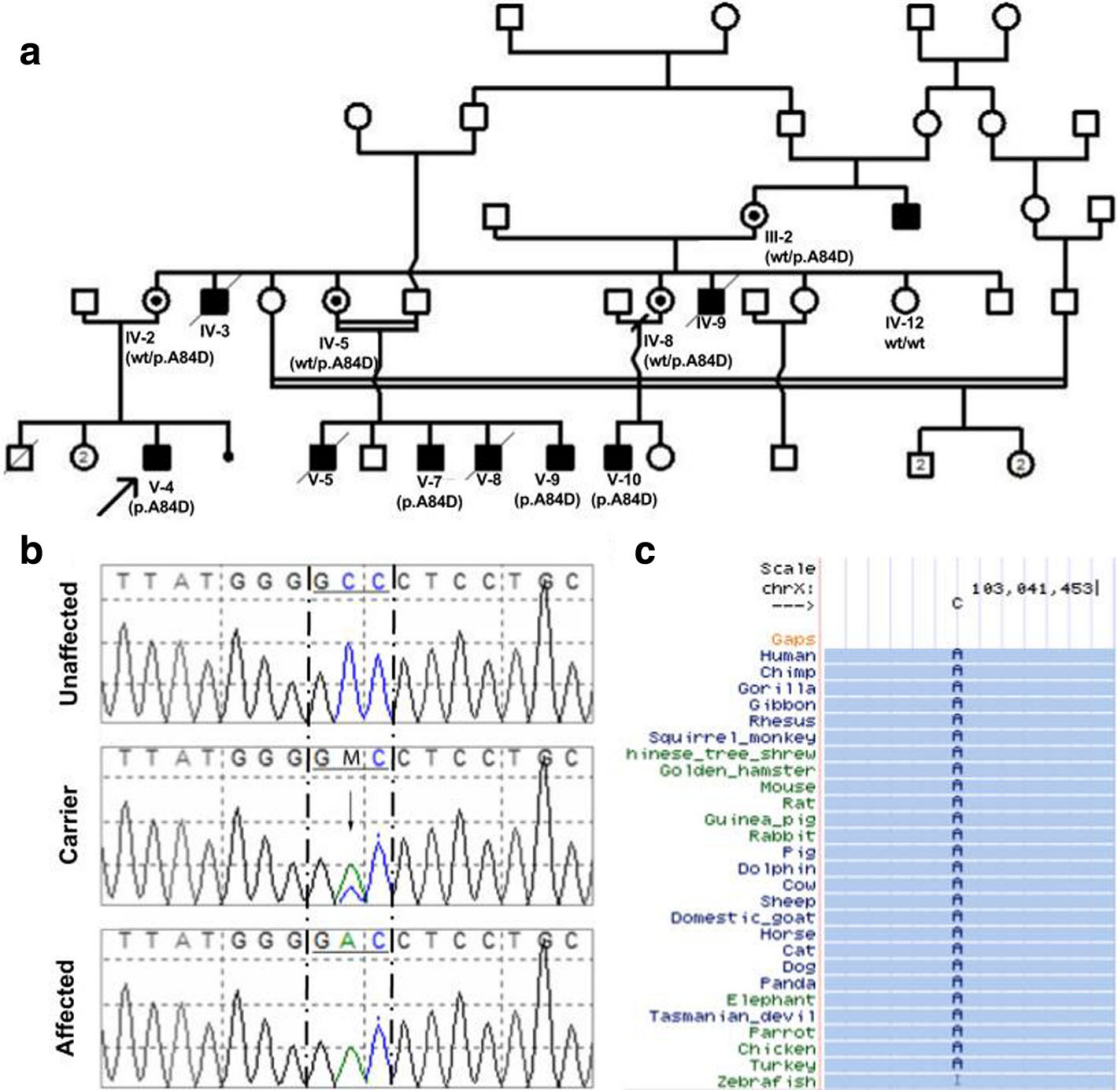
**a** Pedigree of the studied Moroccan family with X-linked epileptic seizures, presenting the co-segregation of the c.251C > A mutation. The patient above the arrow indicates the proband. **b** Chromatograms by Sanger sequencing of family members who are hemizygous, heterozygous and wild-type for the *PLP1* variant. **c** Amino acid conservation map across species demonstrating a highly conserved residues position 84 (based on Multiz Alignments Track of UCSC Genome Browser and The human genome reference hg19/GRCch37)

The propositus (V-4) is a Moroccan 5 years old boy, non-consanguineous, last child of three siblings, and was addressed for familial epilepsy (Fig. 1A). His parents and older sister appear to be healthy and have been free of epilepsy seizure in the past. He had neonatal hypotonia, psychomotor delay, and no seating position with the support of the head at 2 years old. Daily-generalized seizures started at 8 months old and treated by valproate of sodium, but without any response. At clinical examination, he had stature and weight delay at <3rd percentile, microcephaly at -2SD, severe hypotonia, with no facial dysmorphia nor nystagmus. The computerized tomography (CT) scan was normal, but the electroencephalogram (EEG) showed abnormal waves at right fronto-temporal region. Unfortunately, MRI could not be available for the patient.

Five cousins (V-5, V-7, V-8, V-9, V-10) and maternal uncles (IV-3, IV-9) were also followed for epilepsy. IV-3, IV-9, V-5 and V-8 died respectively at four, seven, three and two years. They had all neonatal hypotonia, psychomotor delay and epilepsy beginning at 6 months old.

In an effort to elucidate the causal mutation, we undertook a broader approach by WES on two family members, the propositus V-4 and his cousin V-7. Genomic DNA was isolated from peripheral blood leukocytes using the salting-out method.

The WES was performed at McGill University and Genome Québec Innovation Centre (MUGQIC) on a HiSeq 2000 sequencing instrument (Illumina Inc.). The WES capture was done using SureSelect Human All Exon V4 in solution capture kit (Agilent Technologies) according to the manufacturer’s instructions.

The well-validated GATK software (Genome Analysis ToolKit (GATK) [6] has been used in conjunction with the gold-standard aligner BWA (Burrow-Wheeler Alignment) [7] for assembly. Prior to the alignment, each read was properly assessed and trimmed using various tools from the Picard suite (http://broadinstitute.github.io/picard). Once the alignment was performed, quality of each base has been normalized for each sample, and local realignment for suspected indels region was performed. Variant calling has been performed jointly on all samples processed together using UnifiedGenotyper from the GATK suite. We also used the ANNOVAR software to annotate variants using the most up-to-date public databases (dnSNP, REFSEQ) [8]. The potential functional impact of mutations was predicted using Sorting Intolerant From Tolerant (SIFT), at http://sift.bii.a-star.edu.sg, and Polymorphism Phenotyping 2 (PolyPhen2), at http://genetics.bwh.harvard.edu/pph2/.

The putative mutation was validated via Sanger, sequencing the DNA of 9 family members with the following primers: Forward (AGCCTTGTTAAGGTGCTCGCT) and Reverse (GCTTGATGTTGGCCTCTGGA). Sequencing was performed using BigDye Terminator v3.1 cycle sequencing, and the obtained sequences were analyzed on an ABI 3000 DNA Analyzer (Applied Biosystems).

Informed consent for DNA analysis was obtained from all participants and parents’ patients.

A total of 188,043 variants were detected. The WES data covered all exons of known X-Linked Epilepsy related genes, and no known pathogenic mutations were identified in these genes. Nevertheless, we identified a single hemizygous base substitution in coding exon3, c.251C > A of the *PLP1* gene (based on GRCh37/hg19, and transcript ID NM_000533), resulting in substitution of alanine by an aspartic acid at amino acid 84. This mutation was found in hemizygous state in the propositus V-4 and in his cousin V-7. Sanger sequencing confirmed this mutation in hemizygous state in the affected subjects (V-9, V-10) and in heterozygous state in the obligatory carrier mothers, showing co-segregation of the mutation with the disease phenotype (Fig. 1A). The non-affected IV-12 was wild type for this mutation (Fig. 1B).

Mutation c.251C > A (p.Ala84Asp) located within exon 3, results in changes of a non-polar alanine to a charged aspartic acid at position 84, it was predicted by Polyphen2 to be probably damaging with a score of 0.989 (sensitivity: 0.72, specificity: 0.97). The Mutation was also predicted to be damaging by SIFT with a score of 0. As shown in Fig. 1C, this amino acid change affects highly conserved residues.

## Discussion

The advent and increasingly decreasing cost of NGS and WES has opened up vast new opportunities for its application as a clinical tool to identify mutations in genes established to cause disorders, or in novel genes; altogether facilitating both discovery efforts and conclusive diagnoses [9]. Many studies have demonstrated that systematic application of WES in specific clinical situations has been more accurate, faster, and less expensive than conventional diagnostic procedures [10].

In this study, we report a family showing X-linked connatal severe neurological disorder with epilepsy. WES allowed us to have a conclusive diagnosis identifying a novel missense *PLP1* mutation, suggesting a diagnosis of Pelizaeus-Merzbacher Disease (PMD).

PMD is caused by mutations involving the *PLP1* gene that encodes the myelin protein proteolipid protein 1 (PLP1) and the spliced variant DM20. The c.251C > A mutation, located within the second hydrophobic transmembrane domain of PLP1, has not been previously described. Mutations in the same region of the protein, including c.247G > A and c.254 T > C mutations causing a p.Gly83Arg and p.Leu85Arg amino acid substitutions respectively, have been shown to cause connatal form of PMD [11, 12].

There is a spectrum of *PLP1*-related disorders with some genotype–phenotype correlations [4, 11–13]. Generally, patients with *PLP1* missense mutations show the most severe form of PMD (connatal form), the most common *PLP1* duplications result in the classical PMD, whereas deletions and null mutations in mild form of PMD and SPG2 [4, 11, 13, 14]. Epilepsy, major hypotonia, and severe psychomotor delay were constant features in our affected children and they have the same clinical history. Thus, epilepsy, not being a frequent sign in PMD, represents the specificity of this family. Considering that the majority of *PLP1* point mutations cause more severe dysmyelinating diseases than null mutations, Inoue speculated that the profound dysmyelination resulting from *PLP1* point mutations probably arises not from the absence of functional protein, but rather from the cytotoxic effect of mutant protein [13].

## Conclusion

Our study expands the mutation spectrum of *PLP1* gene and shows that whole-exome analysis in a patient from a family exhibiting X-linked epilepsy could be used efficiently to identify causative mutations, thus providing help to clinicians in making a definitive diagnosis.

## Abbreviations

PLP1: Proteolipid protein 1
PMD: Pelizaeus-Merzbacher Disease
WES: Whole-exome sequencing
