# Correction to: Exome sequencing reveals a novel PLP1 mutation in a Moroccan family with connatal Pelizaeus-Merzbacher disease: a case report

**DOI:** 10.1186/s12887-018-1114-y

**Published:** 2018-04-17

**Authors:** Jaber Lyahyai, Bouchra Ouled Amar Bencheikh, Siham C. Elalaoui, Maria Mansouri, Lamia Boualla, Alexandre DIonne-Laporte, Dan Spiegelman, Patrick A. Dion, Patrick Cossette, Guy A. Rouleau, Abdelaziz Sefiani

**Affiliations:** 1 Centre de Génomique Humaine, Faculté de Médecine et Pharmacie, Mohammed V University in Rabat, Rabat, Morocco; 2 Montreal Neurological Institute and Hospital, Montreal, Quebec Canada; 3 Department of Neurology and Neurosurgery, McGill University, Montreal, Quebec Canada; 4 Département de Génétique Médicale, Institut National d’Hygiène, Rabat, Morocco; 5 Molecular Diagnostic Laboratory and Division of Medical Genetics, CHU Sainte-Justine, Montreal, Quebec Canada

## Correction

After publication of the original article [1] it was brought to our attention that author Bouchra Ouled Amar Bencheikh was incorrectly included as Bouchra Oulad Amar Bencheikh.

The correct spelling of the name is included in the author list of this ‘Correction’ and updated in the original article.
